# Supplementary material for: Injectable pH and Thermo-Responsive Hydrogel Scaffold with Enhanced Osteogenic Differentiation of Preosteoblasts for Bone Regeneration
Source: Pharmaceutics. 2023 Sep 2;15(9):2270. doi: 10.3390/pharmaceutics15092270 (PMC10535719; doi:10.3390/pharmaceutics15092270)
Supplement: Supplementary file 1 [file pharmaceutics-15-02270-s001.zip › pharmaceutics-2578127-supplementary.pdf]

Article

Injectable pH and Thermo-Responsive Hydrogel Scaffold with Enhanced Osteogenic Differentiation of Preosteoblasts for Bone Regeneration

Jasmine L. King [1], Roopali Shirvastava [2], Pooja D. Shah [2], Panita Maturavongsadit [2], and S. Rahima Benhabbour [1,2,*]

[1] Division of Pharmacoengineering and Molecular Pharmaceutics, UNC Eshelman School of Pharmacy, University of North Carolina at Chapel Hill, Chapel Hill, NC 27599, USA; jking116@email.unc.edu

[2] Joint Department of Biomedical Engineering, North Carolina State University and The University of North Carolina at Chapel Hill, Chapel Hill, NC 27599, USA; roopalis@email.unc.edu (R.S.); pdshah1@email.unc.edu (P.D.S.); panita.maturavong@gmail.com (P.M.)

* Correspondence: benhabs@email.unc.edu Tel.: +1-919-843-6142

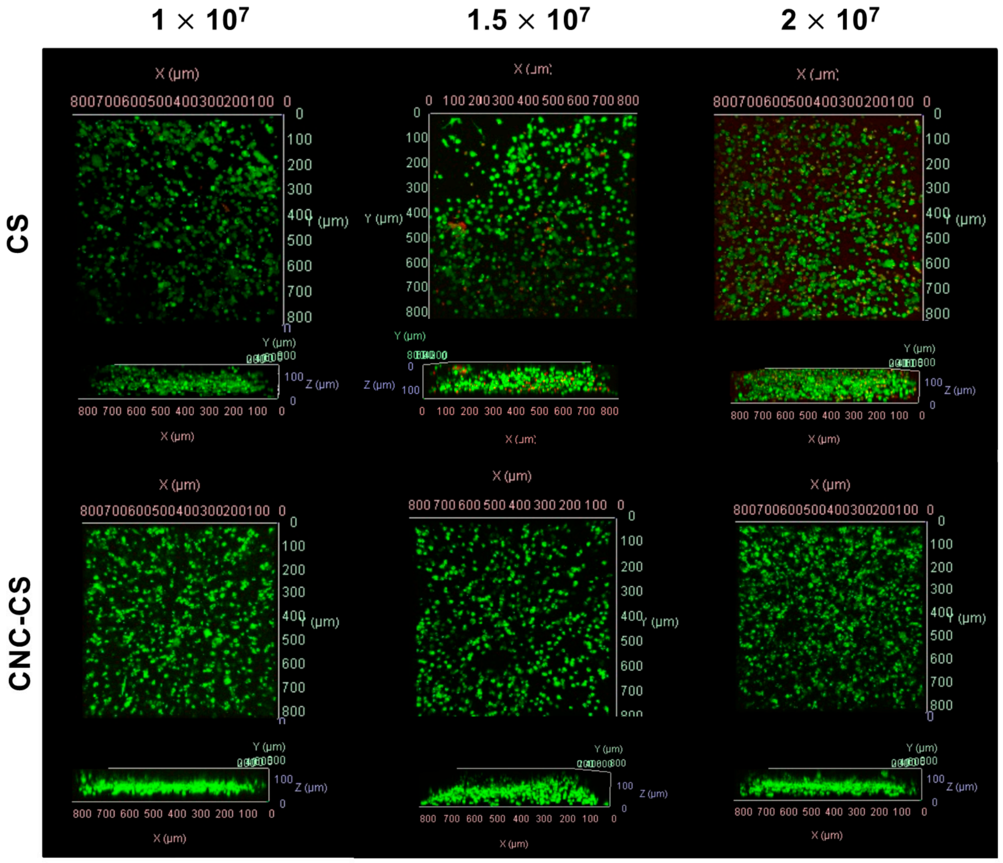

Supplementary Figure S1. In vitro cell viability of MC3T3-E1 CS and CNC-CS hydrogels evaluated after 24 h incubation using LIVE/DEAD® Viability/Cytotoxicity kit. Confocal fluorescent images of MC3T3-E1 cells ($10^7 – 2 \times 10^7$ in CS and CNC-CS hydrogels (n =3; 50 µL samples).

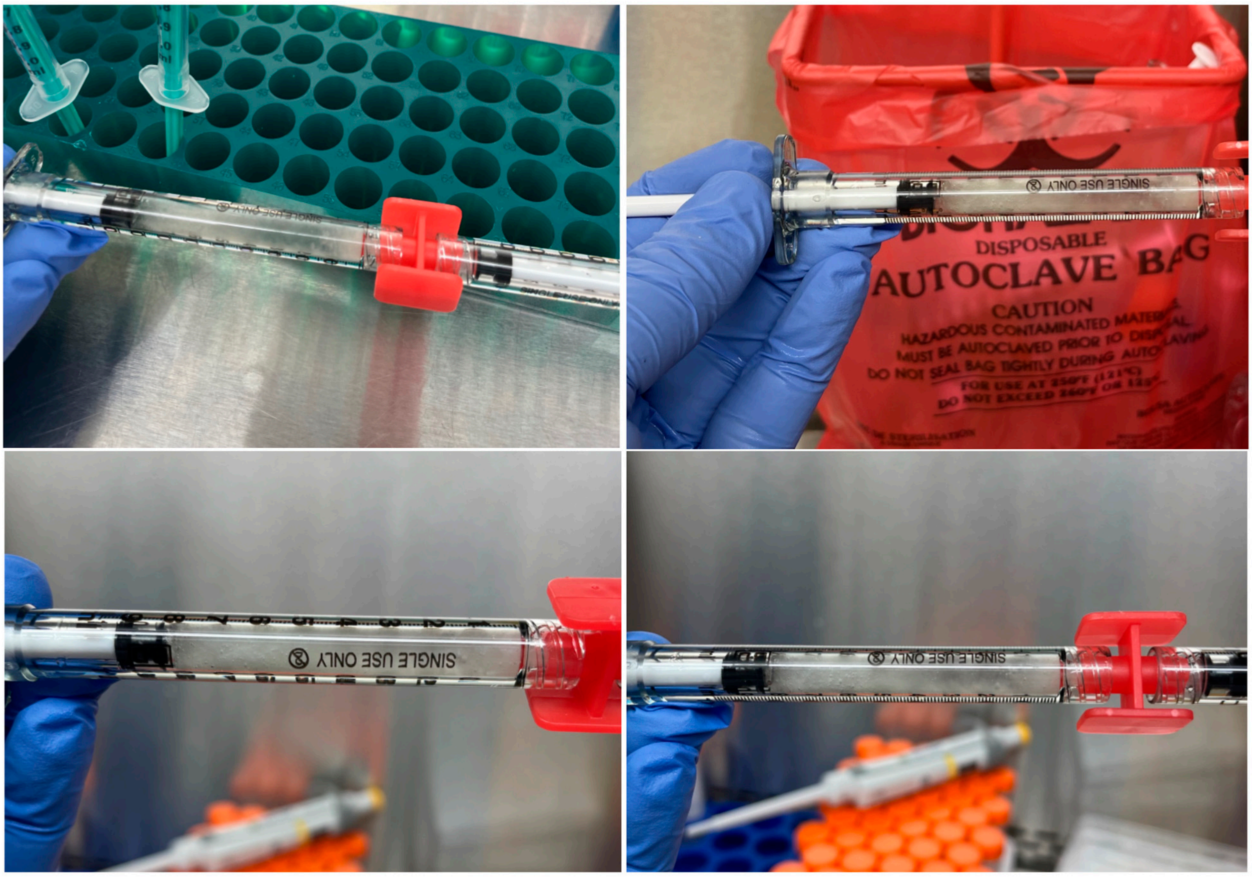

Supplementary Figure S2. Non-uniform distribution of MC3T3-E1 cells within CNC-CS hydrogels at high cell seeding densities. Luer-lock syringes of MC3T3-E1 cells encapsulated in CS and CNC-CS hydrogels at cell densities of $10^7 - 2 \times 10^7$.

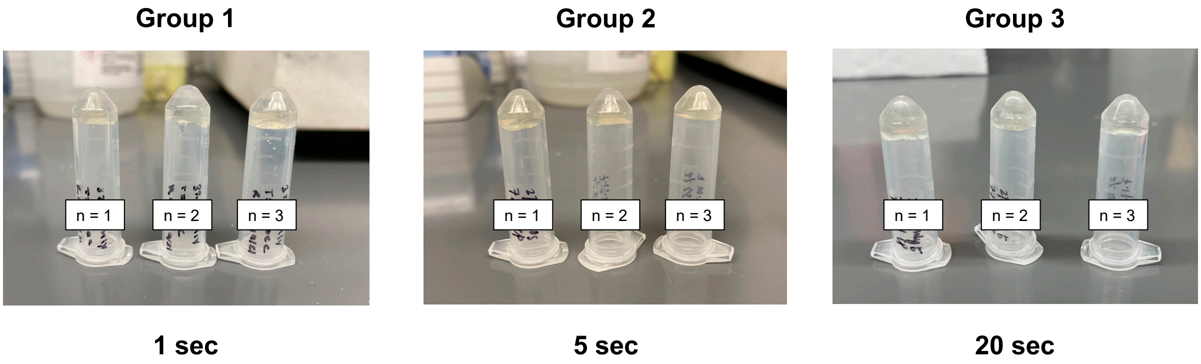

Group	Result (Gelation = Yes; No Gelation = No)
1	No
2	Yes
3	Yes

Supplementary Figure S3. Inversion test for in situ forming CS hydrogel. Inversion test to evaluate hydrogel gelation under temperature-controlled conditions (37°C): Group 1) 1 second at 37°C, Group 2) 5 seconds at 37°C, and Group 3) 20 seconds at 37°C.

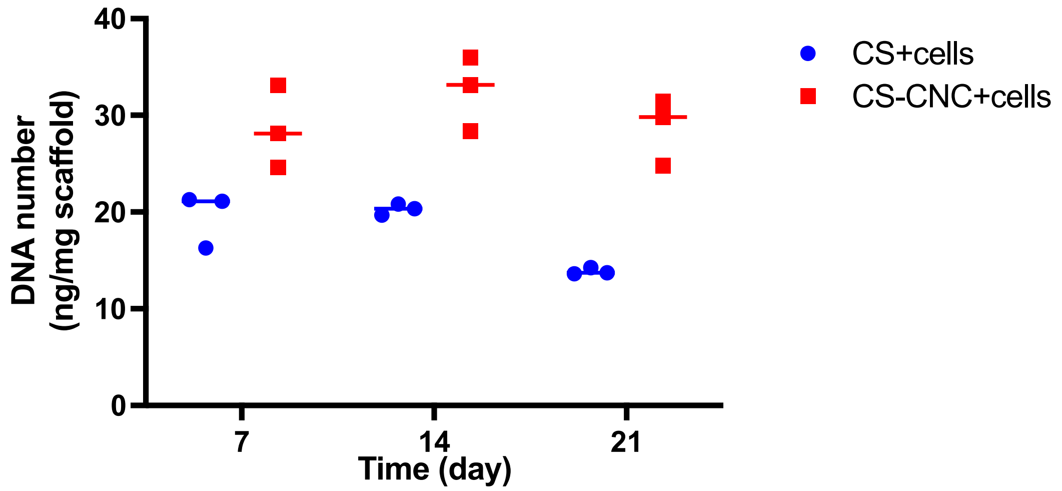

Supplementary Figure S4. Cell proliferation of MC3T3-E1 cells in CS and CS-CNC hydrogels over 21-day study period. Graphical representation of median DNA number of 5 million MC3T3-E1 cells in 1 mL CS and CS-CNC hydrogel.

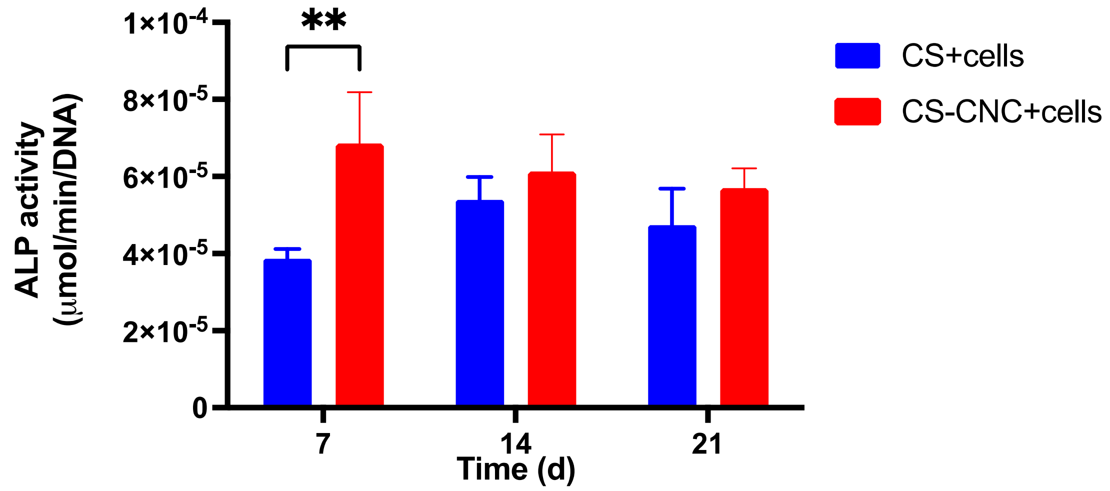

Supplementary Figure S5. ALP activity of cell-laden CS and CS-CNC hydrogels over 21-day study period. Graphical representation of 5 million MC3T3-E1 cells in 1 mL CS hydrogel and CS-CNC hydrogel normalized to DNA number (** indicates $p < 0.005$).

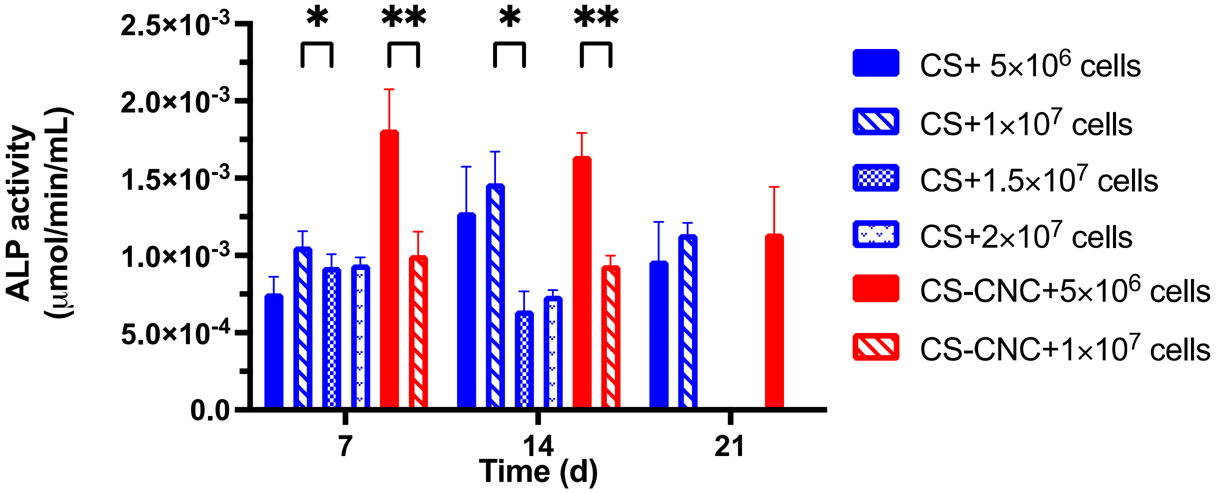

Supplementary Figure S6. ALP activity of cell-laden CS and CS-CNC hydrogels over 21-day study period. Graphical representation of 5, 10, and 20 million MC3T3-E1 cells in 1 mL CS hydrogel and 5 and 10 million MC3T3-E1 cells in 1 mL CS-CNC hydrogel (* indicates $p < 0.05$ and ** indicates $p < 0.005$). There were no intact scaffolds at day 7 for 15 to 20 million CS-CNC hydrogels (no data included).
